# Using Deep Neural Networks to Reconstruct Non-uniformly Sampled NMR Spectra

**DOI:** 10.1007/s10858-019-00265-1

**Published:** 2019-07-10

**Authors:** D. Flemming Hansen

**Affiliations:** Division of Biosciences, Institute of Structural and Molecular Biology, University College London, London, WC1E 6BT UK

**Keywords:** Deep neural networks, NMR, Non-uniform sampling, Sparse sampling, Spectral reconstruction

## Abstract

Non-uniform and sparse sampling of multi-dimensional NMR spectra has over the last decade become an important tool to allow for fast acquisition of multi-dimensional NMR spectra with high resolution. The success of non-uniform sampling NMR hinge on both the development of algorithms to accurately reconstruct the sparsely sampled spectra and the design of sampling schedules that maximise the information contained in the sampled data. Traditionally, the reconstruction tools and algorithms have aimed at reconstructing the full spectrum and thus ‘fill out the missing points’ in the time-domain spectrum, although other techniques are based on multi-dimensional decomposition and extraction of multi-dimensional shapes. Also over the last decade, machine learning, deep neural networks, and artificial intelligence have seen new applications in an enormous range of sciences, including analysis of MRI spectra. As a proof-of-principle, it is shown here that simple deep neural networks can be trained to reconstruct sparsely sampled NMR spectra. For the reconstruction of two-dimensional NMR spectra, reconstruction using a deep neural network performs as well, if not better than, the currently and widely used techniques. It is therefore anticipated that deep neural networks provide a very valuable tool for the reconstruction of sparsely sampled NMR spectra in the future to come.

**Electronic supplementary material:**

The online version of this article (10.1007/s10858-019-00265-1) contains supplementary material, which is available to authorized users.

## Introduction

Reconstruction of non-uniformly sampled (NUS) NMR spectra has become a very important tool for obtaining ultra-high dimensional NMR spectra with high resolution and in a short time. For example, being able to accurately reconstruct NUS NMR spectra allows for high-resolution four-dimensional methyl–methyl NOESY spectra for chemical shift assignment and characterisations of large proteins (Tugarinov et al. 2005; Vuister et al. 1993; Hyberts et al. 2012), five-dimensional spectral of intrinsically disordered proteins for chemical shift assignments (Krähenbühl et al. 2012; Kosiński et al. 2017), and fast characterisation of macromolecular dynamics (Linnet and Teilum 2016), amongst others. Various algorithms have been developed to reconstruct the full dataset from the sparsely sampled data (Hyberts et al. 2012; Ying et al. 2017; Coggins et al. 2012; Orekhov and Jaravine 2011; Balsgart and Vosegaard 2012; Holland et al. 2011; Kazimierczuk and Orekhov 2011), or otherwise extract NMR parameters from the dataset (Eghbalnia et al. 2005; Murrali et al. 2018; Dutta et al. 2015; Pustovalova et al. 2018). Moreover, it has become clear that it is not only the algorithm used to process the sparse data that determines the accuracy with which information can be obtained from NUS NMR data, since the sampling schedule used also has a substantial impact on the final outcome (Hyberts et al. 2012).

The development and application of deep neural networks (DNN) have seen an impressive growth in the last decade with many and highly different applications in all areas of science and technology, including spectroscopy. Traditional applications of DNN include image processing and speech recognition, whereas applications involving the analysis of EPR DEER spectra (Worswick et al. 2018) and reconstruction of MRI (Han and Ye 2018; Hyun et al. 2018) datasets are more recent. Briefly, a supervised DNN contains several *layers* that each transforms an input tensor into an output vector or tensor. For example, a layer can be linear with activation functions, that is, initially the input tensor is multiplied by a parameter-tensor followed by an elementwise mathematical operation, such as tanh(*x*). Training the neural network consist of determining optimal parameters or parameter-tensors and training is therefore a classical minimisation/optimisation problem. Although training a DNN can involve the optimisation of millions of parameters, highly efficient optimisers based on stochastic gradients (Kingma and Ba 2014) have been developed and optimised neural networks now often outperform traditional tools used for analyses.

Reconstruction of NUS NMR data is well suited for supervised DNN, because of the simple architecture of the problem. The input data consists of the sparsely sampled data matrix and a sampling schedule, whereas the output data can consist of the fully sampled NMR spectrum in either the time domain or frequency domain. More importantly, a large training database can easily be generated since the general form of NMR spectra is well known.

It is shown below that a simple DNN based on long short-term memory (LSTM) networks can be trained to reconstruct sparsely sampled one-dimensional NMR spectra. Once the network is trained, the time required for reconstruction is similar to reconstruction times for traditional methods, such as iterative-soft-thresholding (IST) (Hyberts et al. 2012; Holland et al. 2011; Kazimierczuk and Orekhov 2011) and sparse multidimensional iterative lineshape-enhanced (SMILE) reconstruction (Ying et al. 2017). The network developed below is cross-validated using an experimental two-dimensional ^15^N–^1^H NMR spectrum of the 18 kDa T4 Lysozyme recorded with a large sweep width, so that also arginine ^15^N_ε_–^1^H_ε_ signals and are observed. In all cases, the reconstruction with DNN yields reconstructed spectra with small RMSDs to the ‘true’ spectra and more accurate intensities of the resulting peaks in the frequency-domain spectrum compared to traditional algorithms. It is envisaged that with faster computer hardware, reconstruction and analysis of high-dimensional NMR spectra will be substantially improved in the future using deep learning and artificial intelligence.

## Methods

### Training the Deep Neural Network

The DNNs were trained on a standard desktop computer (Intel Core I7-6900 K, 3.2 GHz, 64 GB RAM), equipped with an NVIDIA GeForce GTX 1080 TI GPU graphics card. The *tensorflow* (Abadi et al. 2015) python package with the *keras* frontend was used to generate the network graphs and the optimisation was performed within *tensorflow* using the stochastic ADAM (Kingma and Ba 2014) optimiser with standard parameters and a learning rate of 0.00012. The python *nmrglue* (Helmus and Jaroniec 2013) package was used to read and write NMR spectra in various formats.

Synthetic one-dimensional FIDs were generated using an in-house written *c*++ programme and these FIDs were stored in a binary 2D *nmrPipe* (Delaglio et al. 1995) format. The number of peaks in the synthetic FIDs was randomly chosen between 1 and (3/8)*sp*, where *sp* is the number of sampled complex points in the sampling schedule. Theoretically, two complex numbers or equivalently four real numbers, {intensity, phase, transverse relaxation rate, frequency}, are required to define each signal in the one-dimensional spectrum. The ratio of 3/8, which is slightly smaller than 1/2, was chosen to avoid over-fitting. Thus, for sampling schedules with 32 sampled points a maximum of 12 signals were generated and for sampling schedules with 48 sampled points a maximum of 18 signals were generated. For each signal in each of the synthetic FIDs, an intensity, *I*, a phase, *φ*, a transverse relaxation rate, *R*_2_, and a frequency, ν was randomly chosen. The intensities were randomly chosen between 0 and 1, the *R*_2_ randomly chosen between 3 s^−1^ and 100 s^−1^, and the frequency randomly chosen over the entire sweep-width. Ideally, the phase, *φ*, should be predictable in NMR spectra. However, minor mis-calibrations and off-resonance effects can lead to small deviations from perfectly phased spectra. Therefore, for each signal in the synthetic FIDs, the phase was randomly chosen between − 5^o^ and + 5^o^. In each run of optimisation, a series of 8 × 10^6^ synthetic FIDs were generated (the maximum amount that could be stored in the memory during training). Prior to optimisation, the synthetic FIDs were normalised by the absolute value of the first point.

During each optimisation, 10% of the training data was not included in the optimisation but purely used for cross-validation. For each sampling schedule, the network was trained in runs consisting of 20 epochs until the average mean-squared error for the cross-validation set was less than 5 × 10^−4^ (1.5 × 10^−4^) and the mean-absolute error less than approximately 0.013 (0.008) for 12.5% (18.75%) sampled data. Typically 25–50 runs were needed, which took between 30 and 40 h depending on the specific sampling schedule used.

### NMR Spectroscopy

A two-dimensional ^15^N–^1^H HSQC correlation spectra was recorded on a uniformly ^13^C, ^15^N isotope labelled sample of the L99A mutant of T4 Lysozyme (T4L L99A) that was prepared as described previously (Bouvignies et al. 2011; Werbeck et al. 2013). The NMR spectra was recorded at 25 °C on a Bruker Avance III NMR spectrometer with a ^1^H operating frequency of 700 MHz and equipped with helium-cooled TCI inverse cryoprobe. The fully sampled spectrum was acquired as a 1024 × 256 complex matrix with spectral widths of 12 kHz (^1^H) and 5.1 kHz (^15^N). An adiabatic ^13^C pulse was applied during the ^15^N chemical shift evolution, *t*_1_, for refocussing of ^15^N–^13^C^α^ and ^15^N–^13^CO scalar couplings, and suppression of the H_2_O resonance was achieved using a flip-back pulse (Andersson et al. 1998) immediately after the first INEPT element. Four scans were collected for each *t*_1_ increment with a recycle delay of 1 s resulting in a total experiment time of 34 min.

### Reconstruction of Experimental NMR Spectra

Reconstruction with the iterative-soft-thresholding (IST) algorithm was carried out with the *istHMS* programme (Hyberts et al. 2012) using standard parameters, except that the number of iterations was increased to 1000. The standard parameters were: initial level multiplier, *i_mult* = 0.98, end level multiplier, *e_mult * = 0.98, and correction of first point, *xc  *= 0.5. For reconstruction with the SMILE (Ying et al. 2017) algorithm, the function within *nmrPipe* (Version 2.0 beta Rev 2018.094.15.20 64-bit) was used with standard parameters, except that the noise factor for the signal cutoff (*nSigma*) was decreased to 3.0 from the default value of 5. Running the SMILE algorithm with the default *nSigma* of 5.0 resulted in poor reconstruction in our hands. The standard parameters used for reconstruction with SMILE were: *xP0* = 0.0, *xP1* = 0.0, *zfCount* = 2, *xApod* = SP, *xQ1* = 0.50, *xQ2* = 0.98, *xQ3* = 1.0, *minTDL* = 0.25, *maxTDL* = 4.0, *thresh* = 0.80, *fraction* = 1.0. Prior to analysis, the spectra reconstructed with the SMILE algorithm were divided by the downscaling factor provided in the logfile, so that intensities were comparable with the fully sampled spectrum.

Reconstruction with the DNN algorithm was performed using a python script. First the network graph and the parameters were loaded from the output of the training (see above). Subsequently the reconstructed 1D spectra, one for each ^1^H frequency point, were generated using the *predict* function within *tensorflow*. Finally the reconstructed spectrum was saved in *nmrPipe* format using functions within the *nmrglue* package.

All the reconstructed spectra were Fourier transformed along the ^15^N dimension using the same window function, which was a square-sine window that was shifted by 0.42 π.

### Data Analysis

All NMR spectra were processed using *nmrPipe* (Delaglio et al. 1995) and subsequently analysed using NMRFAM-Sparky (Lee et al. 2015) or in-house written python scripts that utilise functions within the *nmrglue*, *numpy* and *matplotlib* packages. Peak heights were determined using NMRFAM-Sparky and the “Center peak; *pc*” interpolation function.

## Results and Discussion

### A DNN Architecture for NUS Reconstruction

Nuclear magnetic resonance spectra have traditionally been recorded on multi-dimensional regular Nyquist grids, with data recorded at equally spaced time points in each of the dimensions. A multi-dimensional discrete Fourier transform is subsequently used to generate the spectrum in the frequency domain for the identification of signals, their positions, intensities, line-widths and phases. However, as the requirement has increased for higher and higher dimensional NMR spectra, the concept of non-uniform, non-linear, and sparsely-sampled data has been improved substantially since some of its initial applications (Schmieder et al. 1993). Sparse sampling of NMR spectra is possible because the information contained in the spectrum is often far less than the actual number of data points sampled on the full Nyquist grid. For example, for a one-dimensional NMR spectrum each peak is fully characterised by four parameters, the frequency ν, the linewidth Δν = *R*_2_/π, the intentity *I*, and the phase *φ*. Therefore, and in theory, for a one-dimensional spectrum with *n* Lorentzian shaped peaks it is sufficient to sample 4*n* real time-points or 2*n* complex time-points. As the number of dimensions increases the sparseness of NMR spectra typically increases, that is, a smaller fraction of the full Nyquist grid is required to fully characterise all the cross-peaks present. Whereas the linear Nyquist grid is easily processed using the discrete Fourier transform, a linear transformation, processing of sparsely sampled NMR spectra is traditionally more demanding. The major challenge has been to extract the spectral parameters from sparse data because the search-space quickly becomes very large and simple minimisation procedures fail due to the highly non-linear nature of oscillating NMR signals over the non-regular time-domain data matrix.

**Fig. 1:**
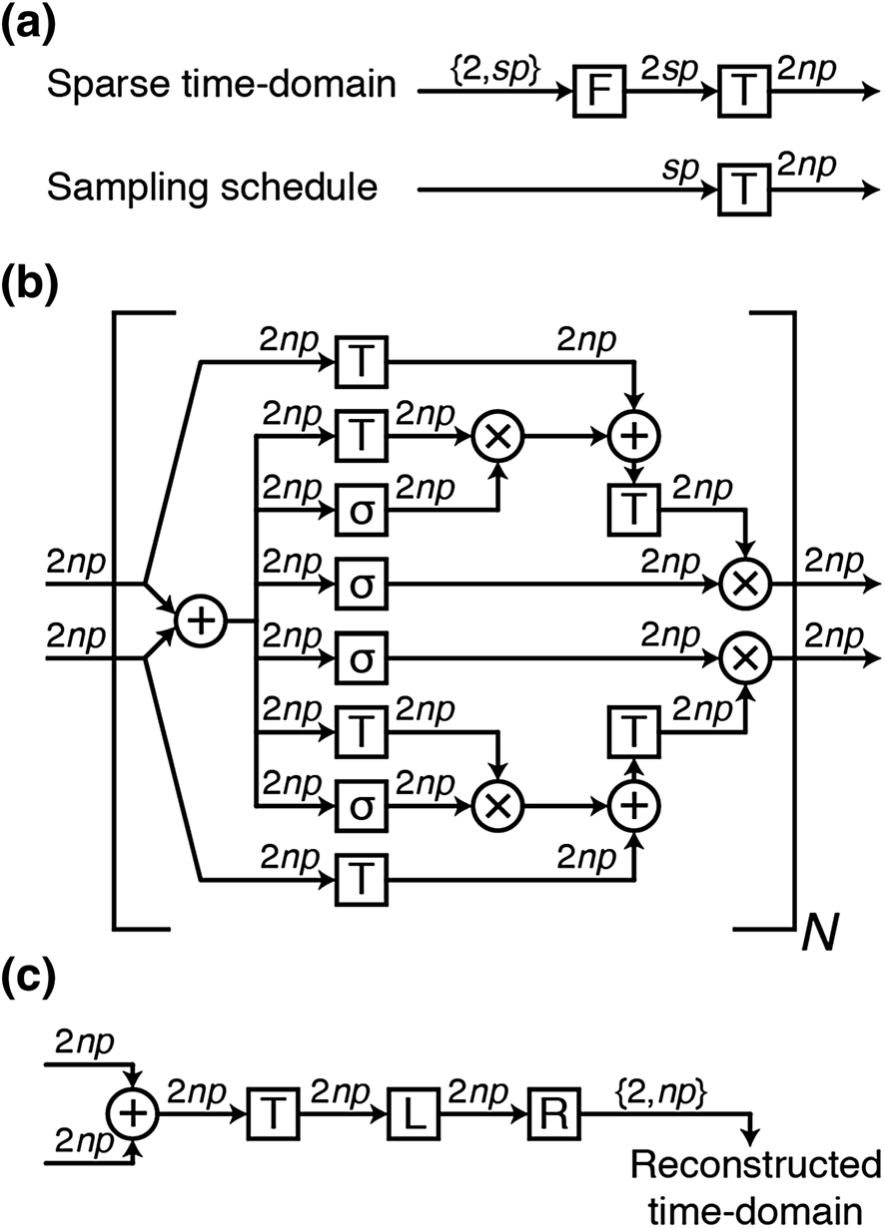
The deep neural network (DNN) architecture and graph developed to reconstruct sparsely sampled NMR spectra. The dimensions of the tensors/vectors are annotated above the arrows. The number of sampled complex points is denoted *sp*, and the number of complex points of the reconstructed spectrum is denoted *np*. **a** The two inputs to the network, the sparse time-domain data and the sampling schedule, are converted to two vectors with dimension 2*np*. Moreover, ‘F’ denotes a flattening layer and ‘T’ denotes a linear layer with tanh(*x*) activation and bias (see text). **b** The modified LSTM cell, where ‘σ’ denotes a linear layer with sigmoidal activation and bias, ‘+’ denotes an elementwise addition layer, and ‘×’ denotes an elementwise multiplication layer. The modified LSTM cell is applied *N* times. **c** The final step of the graph, which takes the two outputs of the last modified LSTM cell as input and produces one output, which is the reconstructed time-domain FID. ‘R’ denotes a reshape layer and ‘L’ denotes a linear layer without activation. (See Supporting Material)

The architecture of the DNN developed here to reconstruct sparsely sampled NMR spectra is shown in Fig. 1. This architecture is designed with inspiration from LSTM networks (Hochreiter and Schmidhuber 1997) that have traditionally been used to analyse time series, for example in finance, handwriting, and for speech-recognition (Chen and Wang 2017; Graves et al. 2009). In a very recent preprint (Qu et al. 2019) an entirely different DNN architecture, based on densely connected convolution neural networks (CNN), has been suggested. Whereas the architecture in Fig. 1 has its roots in the analysis of time series, dense CNN networks are often used for image processing, noise reduction, and removal of artefacts.

For the presented architecture in Fig. 1, the sparsely sampled FID is represented as a 2×*sp* matrix, where *sp* is the number of sampled complex points with one row for each of the real and imaginary data. Initially the sparsely sampled FID and the sampling schedule are transformed into two vectors with length 2*np*, where *np* is the number of complex points in the fully reconstructed one-dimensional spectrum. This transformation is carried out using linear layers with tanh(*x*) activation and bias. Specifically, for a linear layer with the activation function *a*(*x*) and bias **b**, the output vector **y** is calculated from the input vector **x**, as

1 $$\varvec{y} = \left\{ {y_{1} ,y_{2} , \ldots ,y_{n} } \right\} = \left\{ {a\left( {z_{1} } \right),a\left( {z_{2} } \right), \ldots ,a\left( {z_{n} } \right)} \right\}$$

where

2 $${\mathbf{z}} = {\mathbf{Ax}} + {\mathbf{b}}$$

and **A** is the parameter-tensor (kernel). Optimisation of the layer consist of optimising the parameters of the bias **b** and the parameter-tensor **A** (see Supporting Material).

### Training the Deep Neural Network

The DNN was trained separately for each sampling schedule on synthetic data. In each run 8 × 10^6^ fully sampled one-dimensional spectra were generated randomly (see “Methods” section). The input spectra ‘Sparse time-domain’ in Fig. 1a, used for training the DNN were calculated from the fully sampled synthetic spectrum by extracting the points corresponding to the sampling schedule. The cost-function used to optimise the parameters of the DNN was calculated as the average mean-square-derivation between the reconstructed spectra and the fully sampled synthetic spectra. For 12.5% sampled spectra (32/256) the optimisation lead to average mean-squared errors of the cross-validation set of 5 × 10^−4^, showing that the highly non-linear behaviour of the NMR time-domain spectra is well-captured by the deep neural network and the architecture in Fig. 1. Specifically, there is a clear indication that the DNN has indeed ‘learned’ the task of reconstructing the spectra rather than ‘memorising’ and interpolating the training set. For example for 12 peaks, and only considering peak positions, there are 256^12^ = 8 × 10^28^ possible spectra with a resolution of SW/*np*. Additionally, there is differential peak intensities and differential line-widths of the 12 peaks as well as the possibility that less than 12 peaks are present. Thus, the training set far from span the full set of possible spectra, although up to 5 × 10^8^ spectra in total has been used for training,

### Application to an Experimental NMR Spectrum

**Fig. 2:**
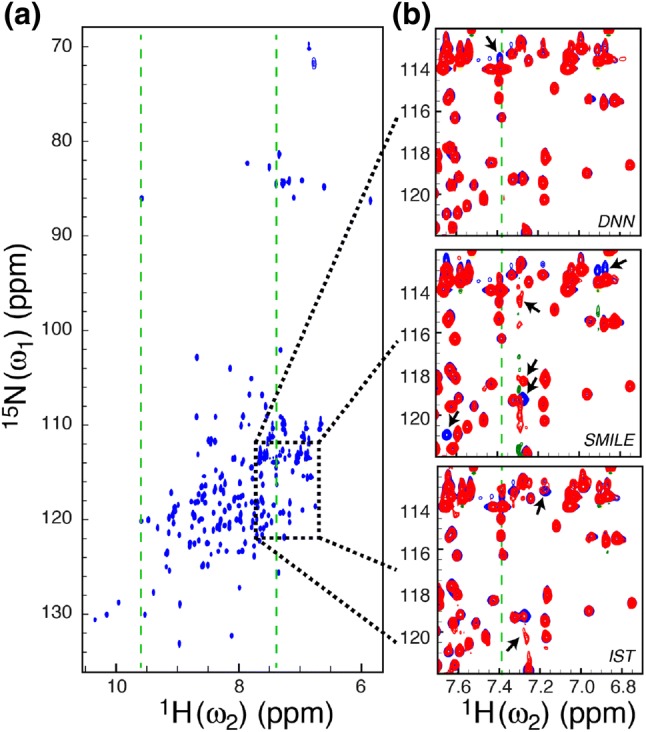
**a** The fully sampled ^15^N–^1^H HSQC spectrum of T4L L99A used to evaluate the performance of the DNN algorithm for reconstruction of sparsely sampled one-dimensional spectra. The peaks between 82 and 88 ppm originate from arginine side-chain ^15^N_ε_–^1^H_ε_. The green vertical dashed lines show where the one-dimensional spectra in Fig. 3 are extracted from. **b** Overlays created for a part of the spectrum (black dotted box in **a**) of the fully sampled spectrum (blue) and the three reconstructed spectra (red), DNN, SMILE and IST. Reconstructed spectra were generated from a sparsely sampled spectrum based on a Poisson-gap sampling schedule with 12.5% of the points sampled (32 out of 256; Table S1). Main differences between the fully sampled spectrum and the reconstructed spectra are indicated with black arrows

A two-dimensional ^15^N–^1^H HSQC correlation spectrum of the L99A mutant of the 164-residue protein T4 Lysozyme (T4L L99A) was used to evaluate the performance of the DNN algorithm and to compared the performance of the DNN algorithm with currently leading algorithms for reconstruction of sparsely sampled NMR spectra. A spectrum with a large ^15^N sweep-width (72 ppm) was recorded with 256 complex points in the ^15^N dimension, such that both the backbone ^15^N–^1^H correlations as well as the side-chain ^15^N–^1^H correlations of arginine and ^15^N–^1^H_2_ correlations of asparagine and glutamine are observed, Fig. 2a. Also, the T4L L99A protein is in chemical exchange (Mulder et al. 2001), which renders some of the ^15^N–^1^H correlations broadened and thereby leading to an even larger range of spectral parameters present in the spectrum.

The experimental ^15^N–^1^H HSQC spectrum was first Fourier transformed in the directly detected ^1^H dimension. Subsequently, for each point in the ^1^H frequency dimension, **f**_H_, the resulting fully sampled ^15^N time-domain spectrum, **t**_N_(**f**_H_) was extracted and the sparsely sampled ^15^N time-domain spectrum, ${\mathbf{s}}_{\text{N}} ({\mathbf{f}}_{\text{H}} )$, was obtained by extracting the points corresponding to the sampling schedule. The reconstructed time-domain spectra, ${\tilde{\mathbf{t}}}_{\text{N}} ({\mathbf{f}}_{\text{H}} )$, were obtained using three different algorithms (*1*) the DNN algorithm presented here, (*2*) the SMILE algorithm (Ying et al. 2017), and (*3*) the hmsIST algorithm (Hyberts et al. 2012). Finally the reconstructed spectra, ${\tilde{\mathbf{t}}}_{\text{N}} ({\mathbf{f}}_{\text{H}} )$, obtained for each of the three algorithms as well as the fully sampled spectrum, **t**_N_(**f**_H_), were Fourier transformed along the ^15^N dimension to generate two-dimensional frequency-domain spectra.

A sparsely sampled spectrum was generated using a 12.5% (32 out of 256 complex points) Poisson-gap sampling schedule (Hyberts et al. 2012). Excerpts of the reconstructed spectra obtained using the three different algorithms, DNN, SMILE and IST, are compared with the fully sampled spectrum in Fig. 2b and Figure S1. Overall, all three algorithms provide a good reconstruction with most of the cross-peaks reconstructed with intensities that are similar to the fully sampled spectrum. The spectrum reconstructed using the SMILE algorithm had artefacts for ^1^H frequencies around 7.3 ppm and some cross-peaks were missing in the reconstructed spectrum.

**Fig. 3:**
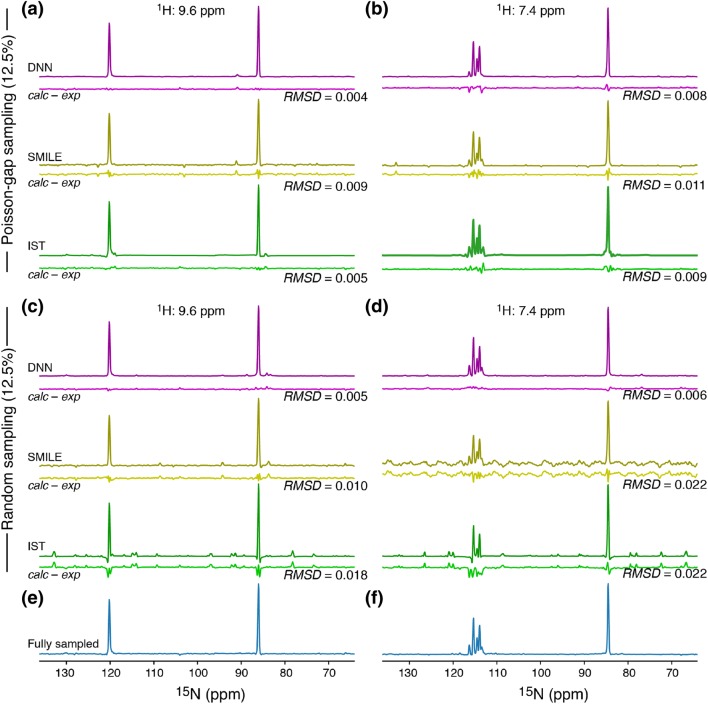
Representative one-dimensional ^15^N slices of reconstructed spectra compared with the corresponding fully sampled spectrum (vertical lines in Fig. 2a). **a**, **c** Reconstructed 1D spectra with a ^1^H frequency of 9.6 ppm and **b**, **d** reconstructed 1D spectra with a ^1^H frequency of 7.4 ppm. **e** and **f** show the corresponding fully sampled spectrum. Spectra in **a** and **b** were reconstructed from a 12.5% Poisson-Gap sampling, while spectra in **c** and **d** were reconstructed from a 12.5% random sampling (Table S1)

To provide a more quantitative comparison, slices along the ^15^N frequency-domain were taken out of the reconstructed spectra and compared to the corresponding slices of the fully sampled spectrum; green vertical lines in Fig. 2a. The first ^1^H frequency, ^1^H of 9.6 ppm, for which a ^15^N slice is shown in Fig. 3 was chosen because it intuitively should be easy to reconstruct due to only two very isolated and sharp peaks being present. The second slice, ^1^H of 7.4 ppm, should intuitively be more difficult to reconstruct since it contains many peaks with differential line widths. A good reconstruction is obtained for the slice at ^1^H of 9.6 ppm, Fig. 3a, c, although reconstruction with the IST algorithm leads to visible artefacts when a random sampling scheme is used. For the more challenging slice, ^1^H of 7.4 ppm, all three algorithms lead to similar RMSDs between the reconstructed and the fully sampled spectrum, Fig. 3b, when a Poisson-gap sampling is used. For the random sampling schedule, Fig. 3d, the DNN algorithm leads to significantly better reconstructions than both IST and SMILE. The fact that the DNN algorithm leads to a significantly better reconstruction for very sparse and random samples is already apparent from a simple visualisation of the reconstructed 2D spectra (Figure S2), where artefacts and extra peaks are observed in the spectra reconstructed with IST in particular.

**Fig. 4:**
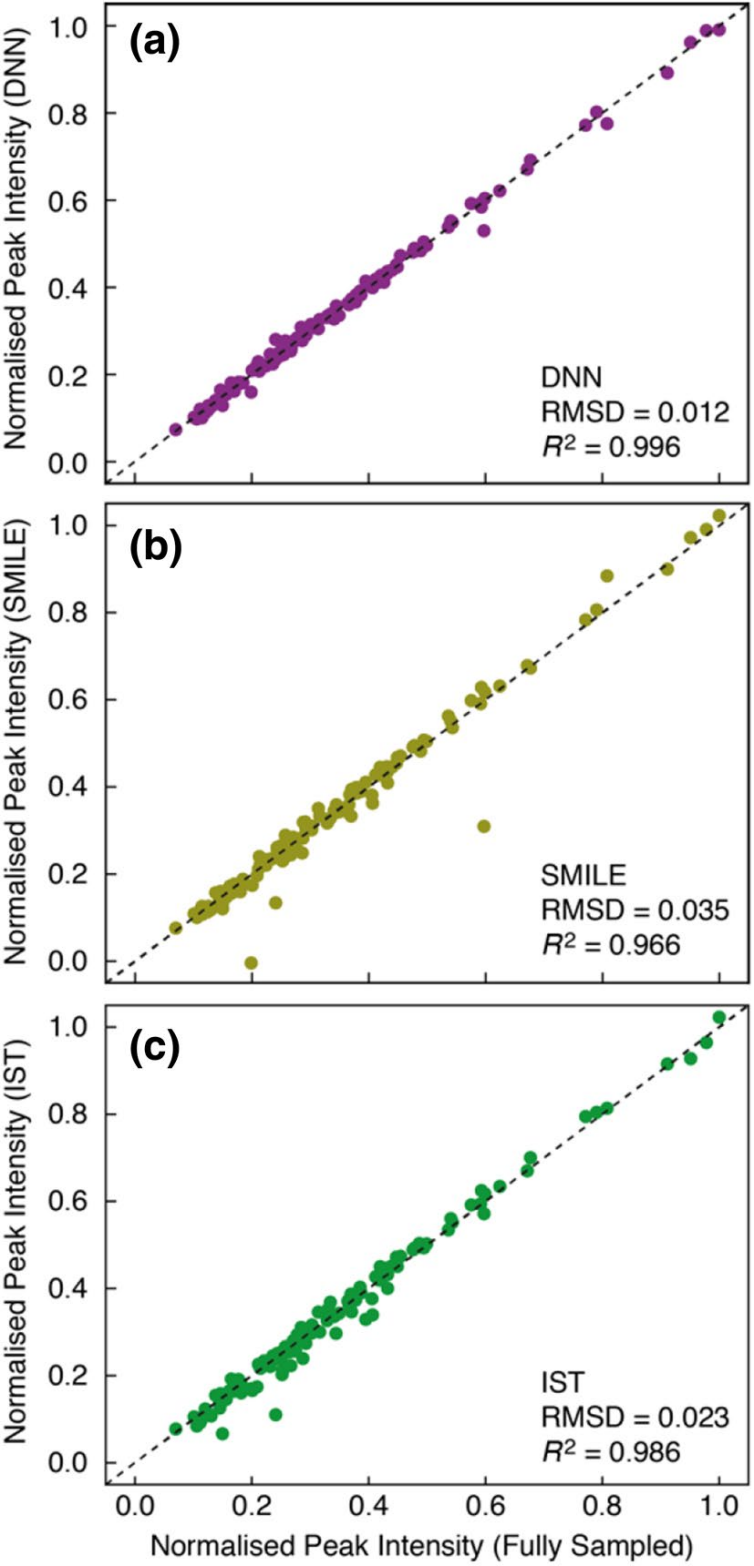
Comparison of normalised peak intensities obtained from the fully sampled spectrum (abscissa axis) with those obtained from reconstructed spectra (ordinate axis). The comparison are shown for **a** reconstruction using the DNN algorithm, **b** reconstruction using the SMILE algorithm, and **c** reconstruction using the IST algorithm. The dashed black line corresponds to *y *= *x* and *R*^2^ is the Pearson coefficient of linear correlation. All reconstructions were carried out on a 12.5% Poisson-gap sampled spectrum (Table S1)

Subsequently, the ability of the different reconstruction algorithms to reproduce peak intensities was quantified. The discrete Fourier transform traditionally used to transform fully sampled spectra is a linear operator as well as an isomorphic transformation. Intensities are therefore represented well in the frequency-domain spectrum. Since the sparse data are sampled on a non-uniform grid, the reconstruction algorithms are inherently non-linear and a quantification of how well peak intensities are reconstructed is therefore important. For a set of 134 isolated peaks, Figure S3, the peak heights were obtained by interpolation (see “Methods” section). Figure 4 shows a comparison of normalised peak intensities obtained from the fully sampled spectrum versus intensities obtained from spectra reconstructed with the three algorithms from a 12.5% Poisson-gap sampling. In this dataset the DNN algorithm had a good overall reproduction of peak intensities with a normalised RMSD of just over 1% and a Pearson correlation between peak intensities measured in the spectrum reconstructed with DNN and the fully sampled spectrum of *R*^2^ = 0.996. This is similar to the Pearson correlation estimated using the densely connected convolution network (Qu et al. 2019), *R*^2^ = 0.992. The SMILE algorithm generally had a good reproduction of peak intensities as well, however, with a handful of peaks rather poorly reconstructed, leading to an overall RMSD of about 3%. The IST algorithm also had a good reproduction of peak intensities, however, with substantial better reconstruction of the more intense peaks and slightly worse reconstruction of weaker peaks, which lead to an overall RMSD of about 2%.

Three types of sampling schedules were evaluated, (*1*) a 12.5% (32/256) random sampling, (*2*) a 12.5% Poisson-gap sampling, and (*3*) a 18.75% (48/256) Poisson-Gap sampling. Three individual sampling schedules were randomly generated for each type leading to a total of nine sampling schedules, Table S1. For each of the three types of sampling schedules the two metrics described above in Figs. 3 and 4 were used to quantify the overall quality of the reconstruction. Firstly, the RMSD between the reconstructed spectra and the fully sampled spectrum was calculated for each of the ^1^H frequency points, as in Fig. 3, and the average RMSD for ^1^H frequencies between 6.5 and 9.6 ppm; 〈RMSD〉_spec_ reported. Secondly, the normalised peak intensities from reconstructed spectra were compared to those obtained from the fully sampled spectrum, as in Fig. 4.

**Fig. 5:**
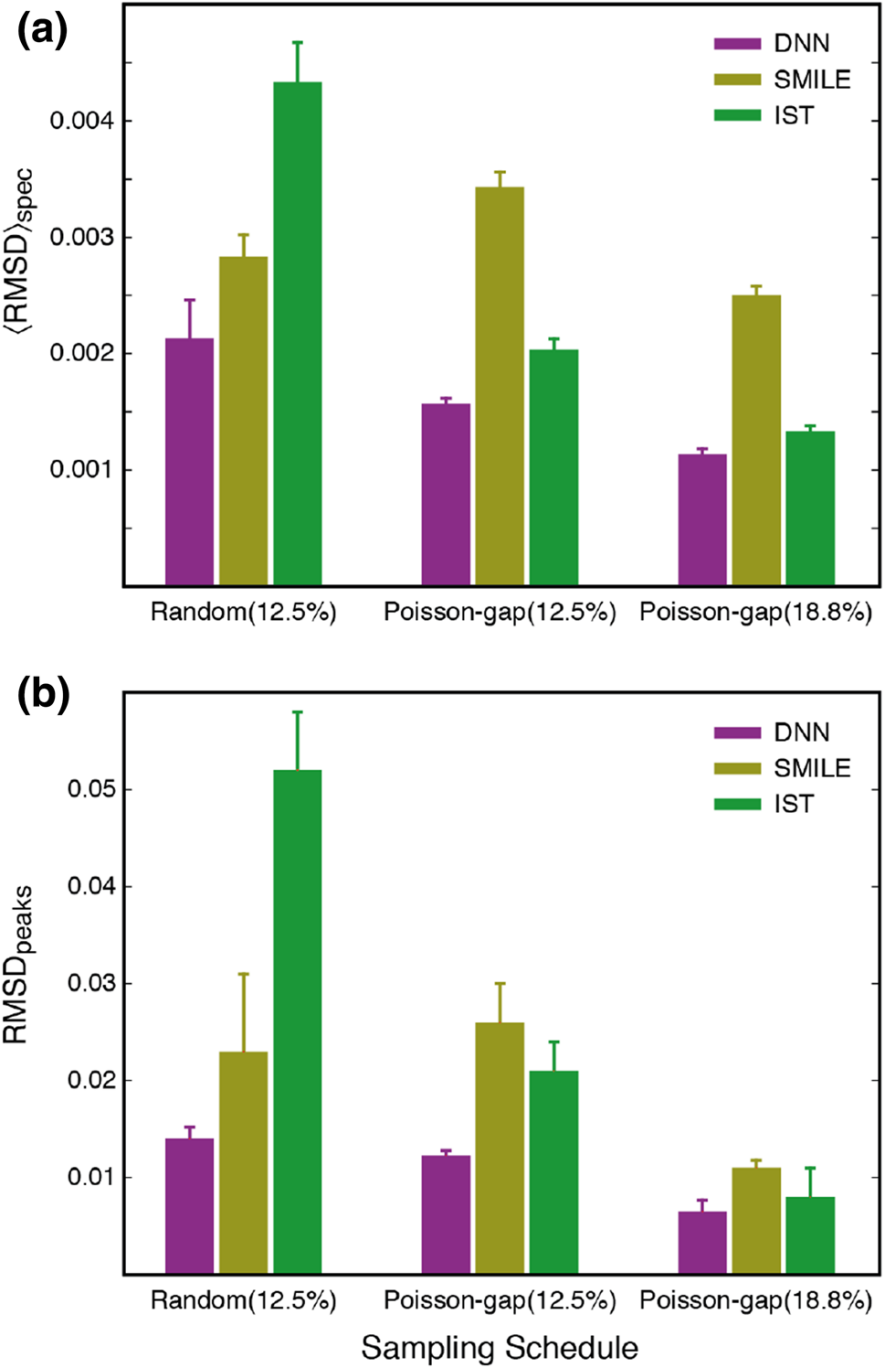
Summary of reconstruction of sparsely sampled one-dimensional NMR spectra. **a** The average normalised RMSD between the fully sampled frequency-domain spectrum and the reconstructed spectrum calculated for ^1^H frequency between 6.5 and 9.6 ppm. **b** The RMSD between normalised peak-intensities obtained from the fully sampled spectrum and the reconstructed spectra. The vertical error-bar shows the standard-deviation for three reconstructions

From the summary in Fig. 5 it is apparent that the DNN algorithm generally leads to better reconstructions for the T4L L99A ^15^N–^1^H spectrum, both in terms of RMSD between the reconstructed spectrum and the fully sampled spectrum as well as reproducing peak intensities. This is particularly the case for the more sparse data (12.5%) and for random sampling schedules. Reconstruction using the IST algorithm improves substantially when a Poisson-gap schedule is used as also pointed out previously (Hyberts et al. 2012).

## Conclusion

Reconstruction of sparse and non-uniformly sampled NMR spectra is increasingly becoming more important as the demand for fast acquisition and ultra-high-dimensional spectra increases. A strategy to reconstruct sparsely sampled NMR spectra using deep neural networks was presented. The proposed strategy employs a new network architecture that is based on LSTM layers, which are frequently used in the analysis of time series. Optimisation of the neural network on a standard desktop computer allowed for excellent reconstruction of sparsely sampled one-dimensional experimental NMR spectra at a level that was as good as, or slightly better than, current algorithms. The time required for reconstruction with the presented neural network is similar to reconstruction times for traditional methods (Hyberts et al. 2012; Ying et al. 2017), albeit longer than an alternative strategy presented very recently (Qu et al. 2019). It is important to stress that in this study deep neural networks were used to reconstruct only one-dimensional spectra, however, the presented strategy shows an avenue for employing deep neural networks to more generally analyse and reconstruct sparsely sampled spectra. It is envisaged that with further explorations of deep network architectures and optimisations, accurate reconstructions of high-dimensional NMR spectra will become possible using deep learning and artificial intelligence.

## Electronic supplementary material

Below is the link to the electronic supplementary material.

Supplementary material 1 (PDF 806 kb)
