# Supplementary material for: Using Deep Neural Networks to Reconstruct Non-uniformly Sampled NMR Spectra
Source: J Biomol NMR. 2019 Jul 10;73(10):577–85. doi: 10.1007/s10858-019-00265-1 (PMC6859195; doi:10.1007/s10858-019-00265-1)
Supplement: Supplementary file 1 — Supplementary material 1 (PDF 806 kb) [file 10858_2019_265_MOESM1_ESM.pdf]

Supporting Material for

Using Deep Neural Networks to reconstruct non-uniformly sampled NMR spectra

D. Flemming Hansen

Institute of Structural and Molecular Biology, Division of Biosciences, University College London, London, United Kingdom, WC1E 6BT

E-mail: d.hansen@ucl.ac.uk

Description of the layers used in the Deep Neural Network:

The layers used for the deep neural network in Figure 1 consist of:

Layer name	Operations
F	A flattening layer denoted 'F'. The flattening layer only re-arranges the data, no calculations are performed, and no parameters are optimised. For a two-dimensional input tensor (a matrix) **x** of size {2, *np*}, the flattening layer returns a one-dimensional vector **y** of length $2 \times np$ that consists of a concatenation of the rows of **x**
R	A reshape layer denoted 'R'. The reshape layer only re-arranges the data, no calculations are performed, and no parameters are optimised. The reshape layer in Figure 1 is used as the reverse of the flattening layer F. Thus, for a one-dimensional input vector **x** of length $2 \times np$ the output **y** is a two-dimensional tensor (a matrix) of size {2, *np*}.
T	A linear layer with hyperbolic tangent, tanh(*x*), activation function and bias denoted 'T'. The layer transforms, in a linear manner, an input vector **x** of size *n* into an output vector **y** of size *m*. This layer contains a two-dimensional parameter-tensor, **A**, with dimension {*m*, *n*} and a one-dimensional parameter-vector, **b**, of size *m*. As described in Eqs. 1 and 2, **y** = $\{\tanh(z_1), \tanh(z_2), \ldots, \tanh(z_m)\}$, where **z** = $\{z_1, z_2, \ldots, z_m\}$ and **z** = **Ax** + **b**. Training the neural network involves optimisation of all the $(n+1) \times m$ parameters of **A** and **b**.
σ	A linear layer with sigmoidal activation and bias denoted 'σ'. This layer is similar to 'T', only difference is that a sigmoidal function is used, $\varphi(x) = (1+\exp(-x))^{-1}$, instead of the hyperbolic tangent.
+	An elementwise addition layer denoted '+'. For two input vectors, **x** and **y**, of identical length *n*, the output vector, **z,** is calculated as: **z** = $\{z_1, z_2, \ldots, z_n\} = \{x_1+y_1, x_2+y_2, \ldots, x_n+y_n\}$.
×	An elementwise multiplication layer denoted '×'. For two input vectors, **x** and **y**, of identical length *n*, the output vector, **z,** is calculated as: **z** = $\{z_1, z_2, \ldots, z_n\} = \{x_1{*}y_1, x_2{*}y_2, \ldots, x_n{*}y_n\}$.

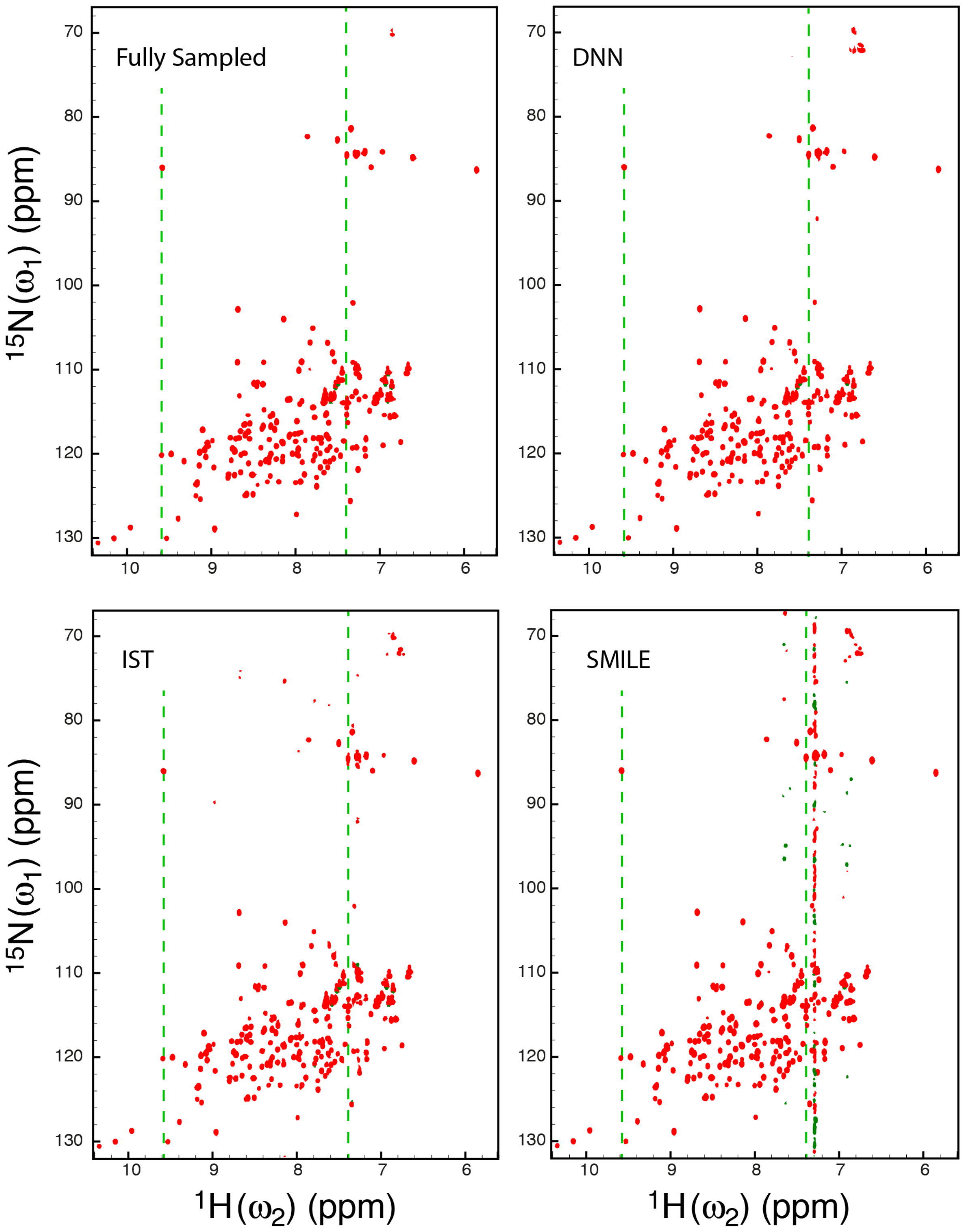

Fig. S1. The ^{15}N-^{1}H HSQC spectrum of T4L L99A used to evaluate the performance of the algorithms for reconstruction of sparsely sampled one-dimensional spectra. Shown is, the fully sampled spectrum as well as the spectra reconstructed from a Poisson-gap 12.5% sampling (Table S1), with the three algorithms DNN, IST[1], and SMILE[2]. The green vertical dashed lines shows where the one-dimensional spectra in Figure 3 are extracted from.

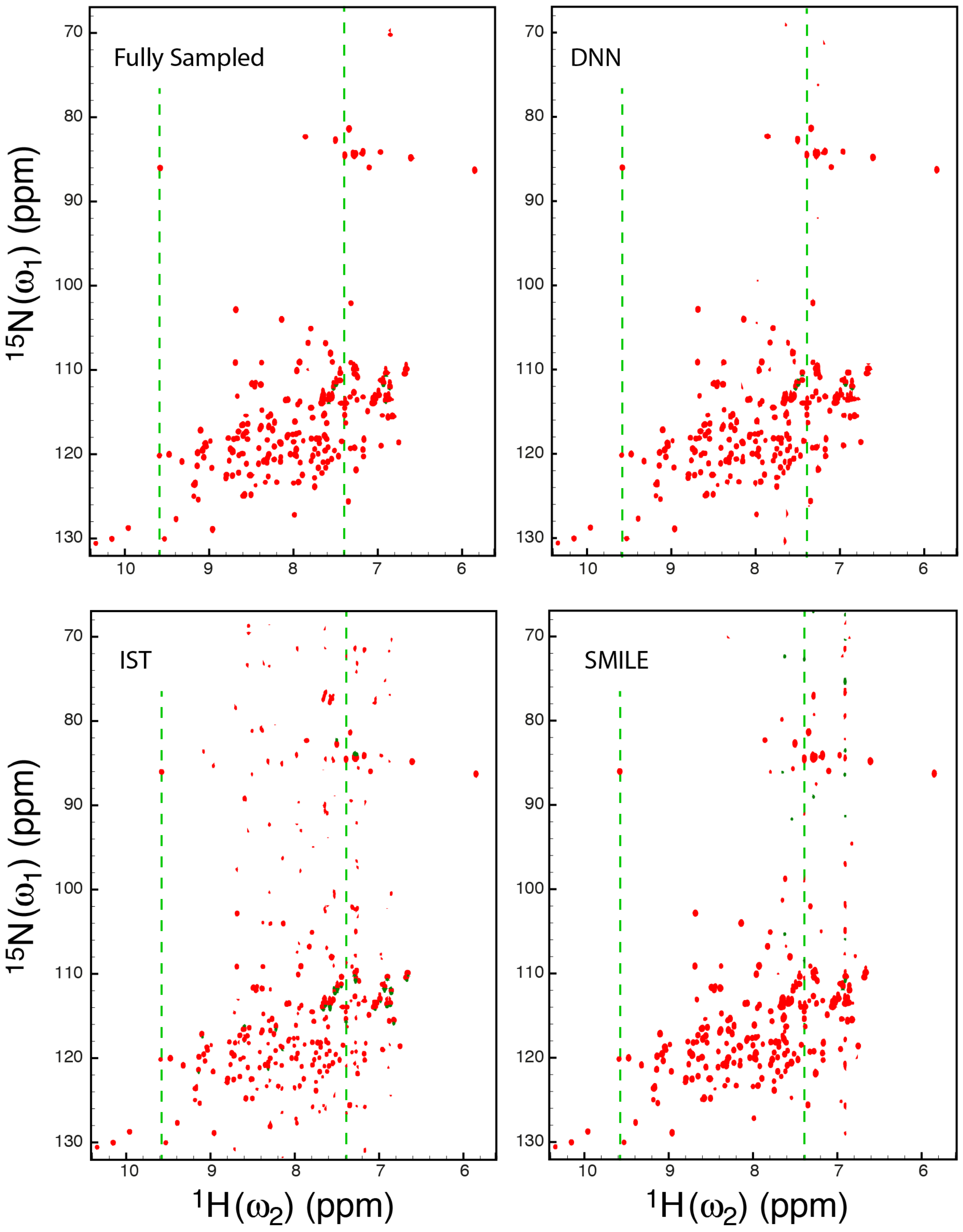

Fig. S2. The ^{15}N-^{1}H HSQC spectrum of T4L L99A used to evaluate the performance of the algorithms for reconstruction of sparsely sampled one-dimensional spectra. Shown is, the fully sampled spectrum as well as the spectra reconstructed from a random 12.5% sampling (Table S1), with the three algorithms DNN, IST[1], and SMILE[2]. The green vertical dashed lines shows where the one-dimensional spectra in Figure 3 are extracted from.

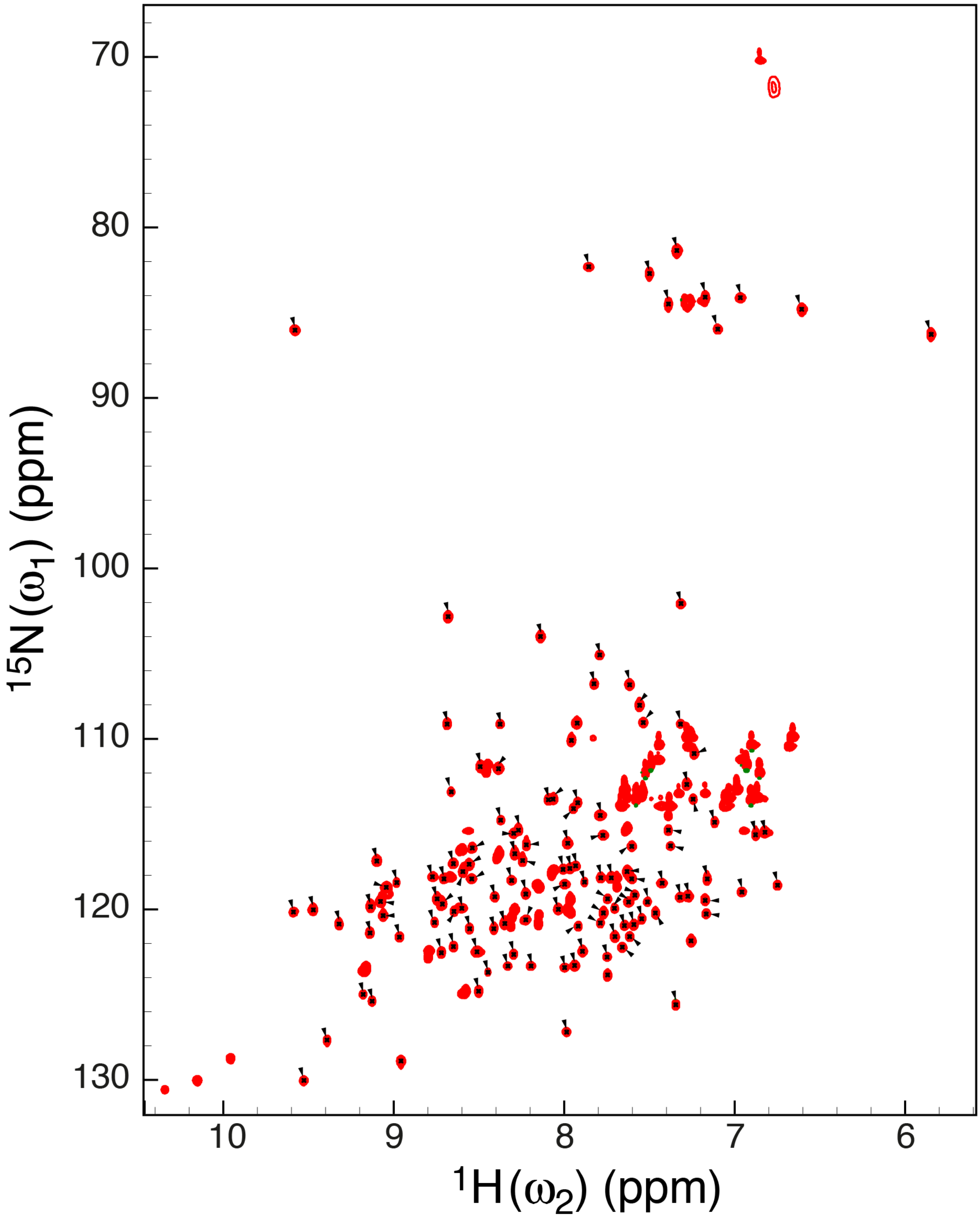

Fig. S3. ^{15}N-^{1}H HSQC correlation spectrum of T4L L99A. Peaks that are not overlapped and used for comparison of intensities, Figure 4 and 5, are shown with black marks.

Table S1. Sampling schedules

12.5% random sampling[a)]			12.5% Poisson-gap[b)]			18.8% Poisson-gap[b)]		
**0**	0	0	**0**	0	0	0	0	0
**12**	11	4	**2**	2	1	1	1	1
**18**	16	9	**3**	3	2	3	2	2
**26**	21	19	**5**	7	3	4	3	5
**28**	31	36	**8**	9	5	5	4	7
**45**	34	42	**11**	12	7	6	5	8
**56**	36	47	**13**	17	10	8	6	9
**77**	41	48	**20**	24	14	9	9	11
**80**	64	56	**30**	28	17	11	12	12
**105**	76	75	**38**	34	20	13	14	15
**106**	77	83	**43**	44	28	15	16	17
**114**	88	98	**55**	54	35	17	20	23
**121**	89	100	**66**	66	41	22	26	29
**132**	93	116	**78**	81	52	26	32	33
**133**	111	117	**93**	97	56	31	38	36
**140**	119	119	**115**	105	72	33	43	40
**142**	120	121	**134**	120	89	40	50	42
**143**	129	132	**151**	143	107	47	59	44
**154**	149	136	**165**	161	131	53	65	50
**160**	156	137	**181**	179	149	57	71	58
**178**	166	138	**192**	192	168	61	81	68
**185**	173	139	**200**	200	192	66	91	74
**193**	180	140	**212**	209	202	73	100	82
**197**	184	143	**227**	220	217	84	110	87
**211**	188	154	**234**	227	229	98	118	96
**221**	195	159	**239**	231	236	106	129	103
**224**	196	185	**243**	238	238	119	143	114
**233**	215	196	**246**	242	242	130	154	122
**240**	218	205	**251**	247	249	148	168	128
**246**	220	239	**252**	250	251	163	175	147
**250**	232	243	**254**	252	252	173	186	161
**251**	254	250	**255**	255	254	188	194	174
						199	204	185
						206	212	196
						211	217	203
						220	224	210
						227	230	218
						234	234	224
						237	236	229
						239	238	235
						241	241	237
						242	243	240
						247	244	243
						249	247	246
						251	249	249
						252	251	251
						253	253	253
						255	255	255

a) Generated using the random function within the python module *numpy*. First schedule, in bold, is used for data in Figure 3c,d and Figure S2. **b**) Generated with the programme provided as a part of the istHMS[1] package using the Knuth algorithm and random seeds of 31415926, 31415925, 31415920, respectively. First schedule, in bold, is used for data in Figures 2, 3a, 3b, and S1.

Supporting References

1. Hyberts, S. G., Milbradt, A. G., Wagner, A. B., Arthanari, H. & Wagner, G. Application of iterative soft thresholding for fast reconstruction of NMR data non-uniformly sampled with multidimensional Poisson Gap scheduling. *J. Biomol. NMR* **52,** 315–327 (2012).

2. Ying, J., Delaglio, F., Torchia, D. A. & Bax, A. Sparse multidimensional iterative lineshape-enhanced (SMILE) reconstruction of both non-uniformly sampled and conventional NMR data. *J. Biomol. NMR* **68,** 101–118 (2017).
